# Methicillin-Resistant* Staphylococcus aureus* Biofilms and Their Influence on Bacterial Adhesion and Cohesion

**DOI:** 10.1155/2016/4708425

**Published:** 2016-12-18

**Authors:** Khulood Hamid Dakheel, Raha Abdul Rahim, Vasantha Kumari Neela, Jameel R. Al-Obaidi, Tan Geok Hun, Khatijah Yusoff

**Affiliations:** ^1^Department of Microbiology, Faculty of Biotechnology and Biomolecular Sciences, Universiti Putra Malaysia, 43400 Serdang, Selangor Darul Ehsan, Malaysia; ^2^Department of Cell and Molecular Biology, Faculty of Biotechnology and Biomolecular Sciences, Universiti Putra Malaysia, 43400 Serdang, Selangor Darul Ehsan, Malaysia; ^3^Institute of Bioscience, Universiti Putra Malaysia, 43400 Serdang, Selangor Darul Ehsan, Malaysia; ^4^Department of Medical Microbiology and Parasitology, Faculty of Medicine and Health Sciences, Universiti Putra Malaysia, 43400 Serdang, Selangor Darul Ehsan, Malaysia; ^5^Agro-Biotechnology Institute Malaysia (ABI), c/o MARDI Headquarters, 43400 Serdang, Selangor, Malaysia; ^6^Department of Agriculture Technology, Faculty of Agriculture, Universiti Putra Malaysia, 43400 Serdang, Selangor, Malaysia

## Abstract

Twenty-five methicillin-resistant* Staphylococcus aureus* (MRSA) isolates were characterized by staphylococcal protein A gene typing and the ability to form biofilms. The presence of exopolysaccharides, proteins, and extracellular DNA and RNA in biofilms was assessed by a dispersal assay. In addition, cell adhesion to surfaces and cell cohesion were evaluated using the packed-bead method and mechanical disruption, respectively. The predominant genotype was* spa* type t127 (22 out of 25 isolates); the majority of isolates were categorized as moderate biofilm producers. Twelve isolates displayed PIA-independent biofilm formation, while the remaining 13 isolates were PIA-dependent. Both groups showed strong dispersal in response to RNase and DNase digestion followed by proteinase K treatment. PIA-dependent biofilms showed variable dispersal after sodium metaperiodate treatment, whereas PIA-independent biofilms showed enhanced biofilm formation. There was no correlation between the extent of biofilm formation or biofilm components and the adhesion or cohesion abilities of the bacteria, but the efficiency of adherence to glass beads increased after biofilm depletion. In conclusion, nucleic acids and proteins formed the main components of the MRSA clone t127 biofilm matrix, and there seems to be an association between adhesion and cohesion in the biofilms tested.

## 1. Introduction

Since it was first identified in 1961, methicillin-resistant* Staphylococcus aureus* (MRSA) has been implicated in nosocomial infections worldwide [1]. These infections can complicate treatments involving in-dwelling catheters and medical implants through biofilm formation [2].

Biofilms can be graded based on the activities of the bacteria within them. Distinct subpopulations of the bacteria are located within the biofilm based on their different metabolic states [3]. The cells on the surface of the biofilm are aerobic, whereas those located deeper, where the oxygen concentration is low, are fermentative and dormant [4, 5]. Therefore, distinct matrix layers representing subpopulations of bacteria are found in biofilms, resulting in different selective pressures and the emergence of antibiotic-resistant strains [6–8]. In most cases, biofilm-associated infections are detected after the biofilms are already formed and can no longer be eliminated because of the tolerance of the biofilm to most antimicrobial treatments [4].

The biofilm matrix components, comprising polysaccharides, proteins, and DNA, play a major role in its general structure and contribute to its conservation and resistance phenotype [9]. In general, two biofilm phenotypes have been identified. Polysaccharide intercellular adhesion- (PIA-) dependent biofilms are composed of poly-*β*-1,6-N-acetylglucosamine- (PNAG-) based matrices. PIA is synthesized from the products of genes located at the* ica* locus [10]. The other type, PIA-independent biofilm, is composed of cell surface components such as teichoic acid [11], fibronectin-binding proteins FnBpA and FnBpB [12–15], and autolysin extracellular DNA (eDNA) [16, 17].

The synthesis of biofilms is influenced by a number of factors. Biofilm production, however, does not appear to be linked to the* ica* locus. O'Neill et al. [18] observed that although the* ica* locus is present and expressed in PIA-independent biofilms, the genes do not appear to be involved in their formation. Houston et al. [19] found that deletion of the major autolysin (*atl*) gene in MRSA strains impaired their ability to form FnBP-dependent biofilms. Some MRSA clinical isolates even produce biofilms of both phenotypes. MRSA strain 132 is able to switch from PIA-dependent to PIA-independent proteinaceous biofilm matrices depending on environmental conditions [15].

The biofilm dispersion is investigated in vitro using enzymatic detachment methods [20] and treatment with chemicals such as periodate (HIO_4_ or NaIO_4_). These conventional methods are used to identify the biofilm matrix components of both Gram-negative and Gram-positive bacteria [21, 22]. Moreover, bacteria within biofilms are significantly affected by matrix components that influence adhesion of the cells to solid substrata and cohesion between bacterial cells [23]. Specific matrix components can increase the ability of bacteria to aggregate [24]. The structure of extracellular polymeric substances (EPS) is complex and variable, and its precise role in cell adhesion and cohesion is not completely understood [17].

The aims of this study were to examine the ability of a collection of MRSA isolates with* spa* type t127 to form biofilms, to determine the extracellular matrix components in the biofilms formed by these strains, and to elucidate the influence of biofilms on the ability of these bacteria to adhere and aggregate.

## 2. Material and Methods

### 2.1. Identification and Genotyping of MRSA Strains

A total of 25 MRSA clinical isolates were obtained from the Medical Microbiology Laboratory at the Universiti Putra Malaysia. These isolates were obtained from different systemic infection sites, and their identity was confirmed by Gram staining, growth on mannitol-salt agar (Oxoid, UK), and CHROMagar MRSA (Paris, France). Kirby-Bauer testing was performed for oxacillin (1 *μ*g) (Oxoid, UK) and cefoxitin (30 *μ*g) (Oxoid, UK) on Muller-Hinton agar (Oxoid, UK) [25]. The MRSA strain ATCC33591 and clinical methicillin-sensitive* Staphylococcus aureus* (MSSA) strain were used as standards in every test, which were performed in triplicate. The isolates were confirmed to be* S. aureus* by detection of the* Sa442* fragment and MRSA by detection of the* mecA *gene. A single polymerase chain reaction (PCR) was used to detect the* Sa442* fragment with the* Sa442* forward primer 5′-AATCTTTGTCGGTACACGATATTCTTCACG-3′ and* Sa442* reverse primer 5′-CGTAATGAGATTTCAGTAAATACAACA-3′. PCR conditions were the following: an initial temperature of 96°C (3 min), followed by denaturation at 95°C (1 min), annealing at 55°C (30 s), and elongation at 72°C (3 min), and a final elongation step at 72°C (4 min). Amplicons of the expected size (108 bp) were obtained [26]. The* mec*A gene was detected using* mec*A forward primer 5′-ACCAGATTACAACTTCACCAGG-3′ and* mec*A reverse primer 5′-CCACTTCATATCTTGTAACG-3′, initial temperature of 95°C (1 min), denaturation 95°C (15 s), annealing 45°C (15 s), and elongation 72°C (30 s), with a final extension at 72°C (4 min). Amplicons of the expected size (162 bp) existed [27]. All isolates were subjected to* spa* typing, according to Christensen et al. [28]. The polymorphic X region of the protein A gene (*spa*) was amplified with primer designed from an* S. aureus* sequence in GenBank (accession number J01786): 1079 F [1079–1099]: 5′-TCATCCAAAGCCTTAAAGACC-3′ and 1516R [1536–1516]: 5′-GTCAGCAGTAGTGCCGTTTG-3′. The PCR reaction was performed using a KOD FX Neo Kit from Toyobo Co., Ltd. (Osaka, Japan) as recommended by the manufacturer. PCR conditions were 94°C for 2 min; 35 cycles each of 94°C for 30 s, 50°C for 30 s, and 72°C for 60 s; and a final extension at 72°C for 5 min. The expected product size was between 300 bp and 600 bp, with the size varying by the number of* spa* repeats. All PCR products were sequenced using 1st BASE (BioSyntech, Inc.) after purification with the GeneJET PCR Purification Kit (Thermo Fisher Scientific). Sequence assembly was performed in Clone Manager Basic 9 (SciEd), followed by analysis of the* spa* tandem repeats using* spa* typing online software (http://spatyper.fortinbras.us/) and the Ridom Spa Server database (http://www.spaserver.ridom.de/) [29].

### 2.2. Biofilm Semiquantification with Crystal Violet (CV) Staining

Biofilm formation by MRSA strains was quantified using the microwell plate method described by Christensen et al. and Manago et al. [28, 29]. All MRSA isolates were grown in tryptone soya broth with 1% glucose (TSBG), and then 250 *μ*L of each bacterial strain culture was diluted to an *A*_600_ of 0.05 and incubated in 96-well flat-bottomed polystyrene microwell plates (MWP) at 37°C for 48 h without shaking. The well contents were removed by flipping the plates, and the wells were washed with phosphate buffered saline (PBS, pH 7.2), heat-fixed by exposing the plate to hot air at 60°C in a hybridization oven (model HS-101, Amerex, USA) for 1 h, and then stained with 250 *μ*L of 0.1% (w/v) CV solution for 15 min at room temperature to allow the dye to penetrate the biofilm and be washed with tap water. The plates were emptied and left to dry overnight. Biofilms were quantified by eluting the CV stain with 250 *μ*L of 33% glacial acetic acid and measuring the absorbance of the solution at 570 nm (*A*_570_) using a BioTek Synergy 2 plate reader. The biofilm assay was performed for each strain in triplicate using a microwell plate, and the background was determined by using noninoculated media as a control. The amount of biofilm produced was quantified by comparing the experimental values between the inoculated and noninoculated media. The cut-off value of noninoculated media at an optical density at 570 nm (OD_570_) was recorded as 1.31. This value was considered the deadline point to define biofilm quantities. The biofilm formation abilities of isolates were categorized based on this value. The isolates were considered strong biofilm producers and denoted as “+++” when the absorbance was more than 5.24 (*A*_570_ > 5.24), moderate biofilm producers as “++” when the absorbance was between 2.62 and 5.24 (*A*_570_ = 2.62–5.24), weak biofilm producers as “+” (1.31 < *A*_570_ < 2.62), and biofilm nonproducers as “−” (*A*_570_ < 1.31). These criteria were established by Stepanović et al. [30].

### 2.3. Phenotypic Evaluation of Colony Morphotypes

Colony morphologies were assessed using a spot test on tryptone soya agar (Oxoid, UK) supplemented with 1% glucose (TSAG), whereas Congo red agar [brain heart infusion agar (Oxoid, UK) supplemented with 5% sucrose and 40 *μ*g/mL Congo red dye (BDH Chemicals Ltd., UK)] was used to differentiate between PNAG-producing (black colony) and non-PNAG-producing (red colony) phenotypes as described previously [18]. In brief, strains were cultured on TSAG (1% glucose) plates at 37°C for 16 h. Cells were resuspended in tryptone soya broth (TSB) medium, and the concentration was adjusted to an OD_600_ of 2. Five microliters of the suspension was spotted on TSAG and Congo agar plates. The phenotype was observed after 48 h.

### 2.4. Biochemical Composition of Biofilms

Biofilms were prepared in 96-well plates of MWP as described above and then treated with 250 *μ*L of 40 mM NaIO_4_ in 50 mM sodium acetate buffer (pH 5.5) for exopolysaccharides degradation; proteinase K (0.1 and 1 mg/mL) in 20 mM Tris-HCl (pH 7.5) with 100 mM NaCl and trypsin (0.1 and 1 mg/mL) for protein degradation; 140 U/mL DNase I in 5 mM MgCl_2_ for DNA degradation; and RNase 100 *μ*g/mL for RNA degradation. All plates were incubated for 16 h at 37°C, except for plates with NaIO_4_ and its control, which were incubated at 37°C in the dark for 16 h [22, 31, 32]. In addition, deoxyribonuclease with a final concentration of 140 U/mL in magnesium peptone water buffer (0.1% peptone and 5 mM MgCl_2_), which was incubated at 37°C for 16 h, and proteinase K with a final concentration of 100 *μ*g/mL in Tris-peptone buffer (0.1% peptone, 20 mM Tris-HCl [pH 7.5], and 100 mM NaCl), which was incubated at 37°C for 16 h, were added successively to the established biofilm in MWP. Control wells were filled with appropriate buffers without enzymes. The biofilms were rinsed twice with PBS (pH 7.2), dried for 1 h at 60°C, and stained with 0.1% CV as described above. Biofilm dispersion was calculated as the absorbance of the CV-stained biofilm at 570 nm. For each sample, three replicates were used, and each experiment was repeated at least three times independently.

### 2.5. Role of Biofilms in MRSA Adhesiveness and Cohesiveness

**Figure 1:**
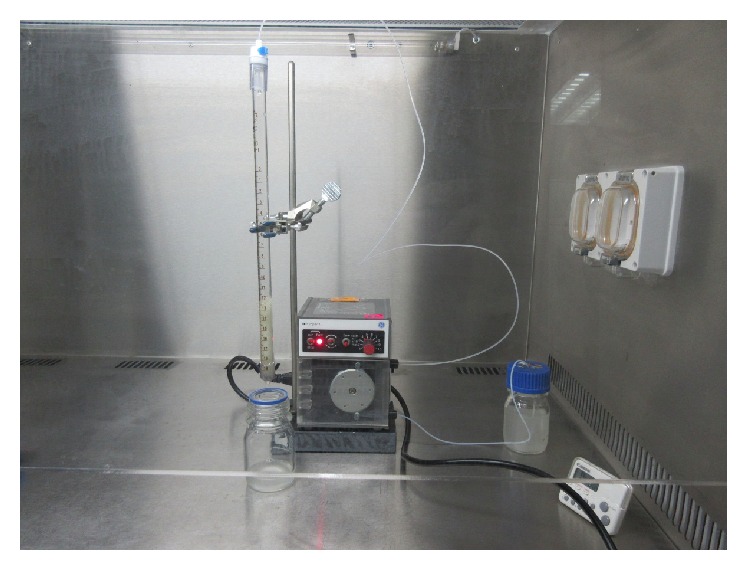
The packed-bead method was used to test cell adhesiveness.

Two preparations of bacterial cells, “unwashed cells” and “washed cells,” were prepared for each MRSA isolate. After an overnight incubation in TSB supplemented with 1% glucose, each bacterial culture was diluted to OD_660_ = 0.8 in TSB without glucose. Then, 80 mL from each sample was centrifuged at 8000 ×g for 10 min. The pellet formed was dissolved in 80 mL PBS (pH 7.2). These cells were considered “unwashed cells”; a substantial amount of biofilm matrix was left on their cell walls. Mechanical disruption was used to prepare “washed cells” by repeatedly dissolving cell pellets in 80 mL PBS (pH 7.2), followed by sonication (Sonic Ruptor 400, OMNI International, GA, USA) for 5 min (1 min sonication, power output 5, pulses 5, with 30 s rest) and centrifugation. The supernatant was discarded, and the cell pellet was resuspended in PBS by vortexing. This process was repeated five times. Washed and unwashed cells of each of the 25 bacterial isolates were used to determine cell adhesiveness by the packed-bead method as shown in Figure 1 according to [24].

MRSA biofilm cohesiveness (aggregation) was assessed using the washed cells. Total culture turbidity was measured at 660 nm, with the initial turbidity designated OD_*t*_ and the culture after the fifth round of sonication designated OD_*s*_. The percentage of cells that were aggregated was estimated as follows: % aggregation = [(OD_*t*_ − OD_*s*_) × 100]/OD_*t*_, as described previously [33, 34]. These experiments were performed three times independently in a sterilized laminar flow cabinet.

## 3. Statistical Analysis

All statistical analyses were performed using SPSS Statistics 21 for windows (IBM). Data were expressed as mean values ± standard error of mean (SEM). Comparison of OD_570_ and OD_660_ between groups was carried out using Student's *t*-test. All results were considered statistically significant at the *p* < 0.05 level.

## 4. Results

### 4.1. Confirmation of* S. aureus* Identity

**Figure 2:**
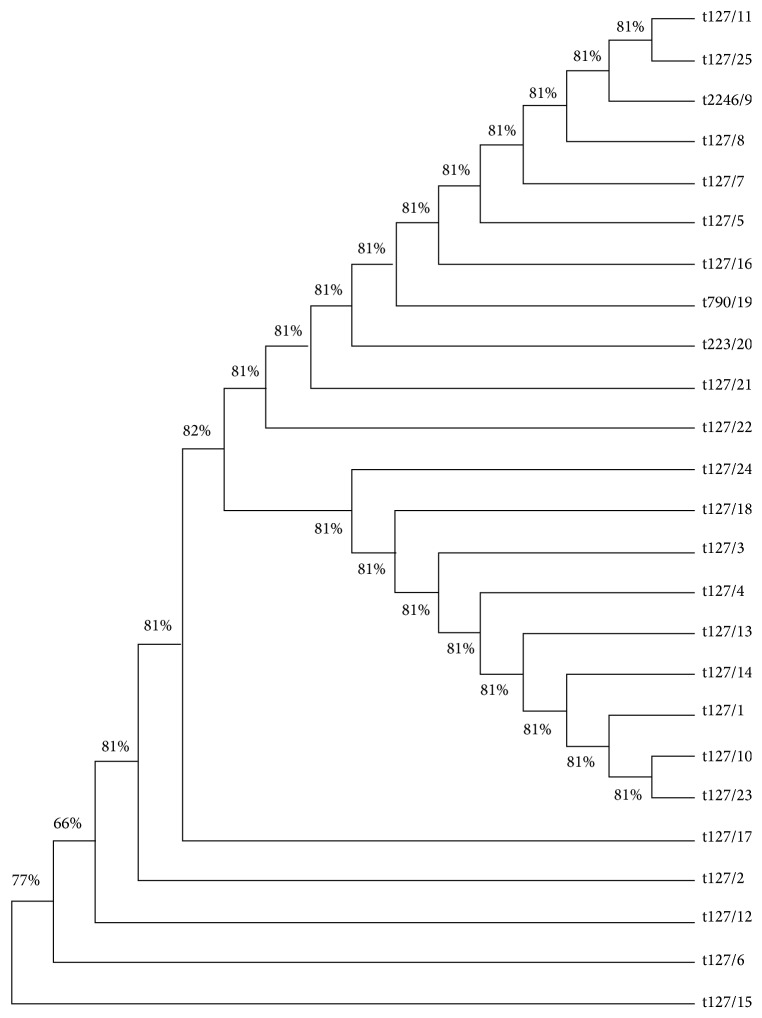
Phylogenetic tree based on the* spa* type of MRSA isolates. The phylogenetic tree was constructed based on the nucleotide sequences of* spa* gene using MEGA7: Molecular Evolutionary Genetics Analysis version 7.0 by using neighbour-joining method.

All isolates studied produced golden-yellow, round, smooth, raised, and mucoid colonies surrounded by a large yellow zone on mannitol-salt agar and changed in colour from rose to mauve on CHROMagar MRSA. These isolates were confirmed to be* S. aureus* by the presence of the specific glutamate synthetase (*Sa442*) fragment and to be methicillin-resistant by the presence of the* mecA* gene. All isolates were completely resistant (100%) to oxacillin and cefoxitin. Isolates were classified into four clones, with the majority (22/25) belonging to clone t127, and the others belonging to t2246 (1/25), t790 (1/25), and t223 (1/25). Phylogenetic tree analysis for these clones was shown in Figure 2. Furthermore, the Ridom Spa Server-Spa-MLST mapping shows that clone t127 related to sequence type (ST-1).

### 4.2. Biofilm Formation

Of the 25 MRSA isolates, 22 (88%) exhibited moderate biofilms with an average OD_570_ ranging from 2.696 to 3.257, whereas three (12%) exhibited weak biofilms with an average OD_570_ between 1.916 and 2.590. The vast majority of the isolates (19/22) belonged to clone t127 and exhibited moderate biofilm formation (Table 1).

### 4.3. Morphology of MRSA

**Figure 3:**
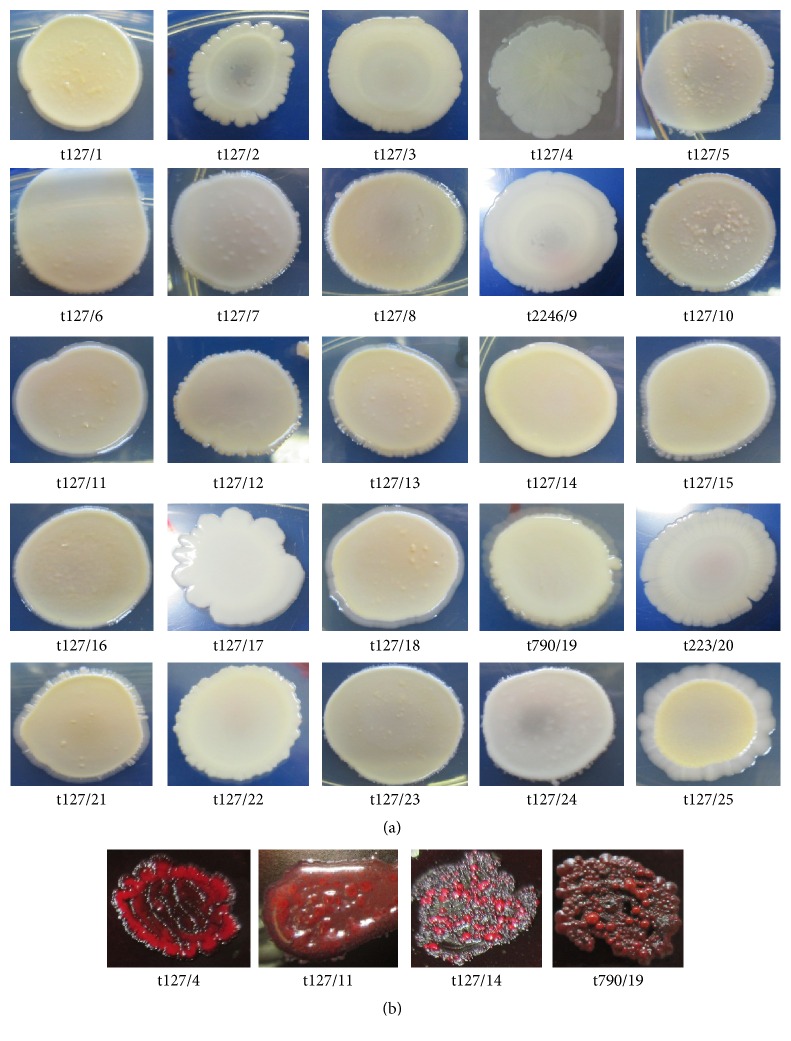
Colony morphologies as distinguishing features of methicillin-resistant* Staphylococcus aureus* biofilms. (a) Morphology of colonies produced on TSA supplemented with 1% glucose. Most colonies had the same structure in the middle, with a wide, circular, and smooth appearance (t127/14, t127/17, t790/19, t223/20, and t127/22), whereas other isolates (t127/1, t127/2, t127/3, t127/5, t127/6, t127/7, t123/8, t127/10, t127/11, t127/12, t127/13, t127/15, t127/16, t127/16, t127/18, t127/21, t127/23, t127/24, and t127/25) showed net-like structures with small, raised nodules in transparent layers with irregular margins. Clones t127/2, t2246/9, and t223/20 showed unique structures with large cavities in the middle surrounded by highly autoaggregated transparent cell layers. Isolate t124/4 formed colonies that appeared like transparent flowers, with circular and vertical lines radiating from the centres of the colonies. (b) Morphology of colonies produced on Congo red agar (CRA) medium; differences based on biofilm components can be seen. The interaction of proteins with Congo red dye produced a red colour, whereas a black colour resulted from the interaction of the dye with exopolysaccharides. Images were captured by a digital camera (Canon IXUS265 HS).

MRSA biofilms on TSA with 1% glucose developed complex architectural features as shown in Figure 3(a), including a layer of highly autoaggregated cells at the centre of each colony, mounted on transparent layers of adherent cells with irregular margins along the edges. Some colonies had circular or vertical lines radiating from the centre, giving the colonies a bloom-shaped appearance. Some of these colonies were black because of the presence of exopolysaccharides or red because of the presence of proteins on Congo red agar (Figure 3(b)).

### 4.4. Biofilm Components

**Figure 4:**
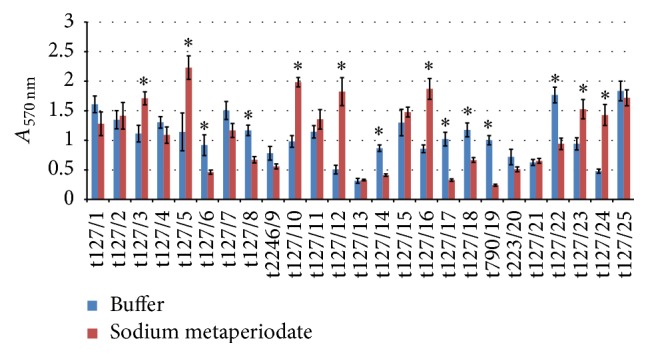
Dispersal of 48 h mature methicillin-resistant* Staphylococcus aureus* (MRSA) biofilms by NaIO_4_. The MRSA isolates are indicated on the *x*-axis; the biofilms matured for 16 h were treated by buffer alone (blue bar) or buffer containing 40 mM/mL NaIO_4_ (red bar). Bars represent the mean values ± standard error of the mean of at least three independent experiments. Asterisk (*∗*) indicates a *p* value of less than 0.05 between the treated group and corresponding control.

The mature MRSA biofilms were examined for interactions with NaIO_4_, proteinase K, trypsin, DNase I, and RNase A.  Figure 4 shows 48 h MRSA biofilms formed in microwell plates that were subsequently exposed to NaIO_4_ for 16 h. Some isolates showed significant detachment of biofilms and displayed reductions in biofilms of 76% (t790/19), 67% (t127/17), and 42–52% in the rest of the isolates. In contrast, isolates t223/20, t2246/9, t127/7, t127/25, and t127/1 showed only a slight reduction in biofilm formation in the presence of NaIO_4_. The remaining isolates (t127/3, t127/5, t127/10, t127/16, t127/23, and 127/24) showed an increase in biofilm formation when treated with NaIO_4_ of up to twofold compared to that of the control.

**Figure 5:**
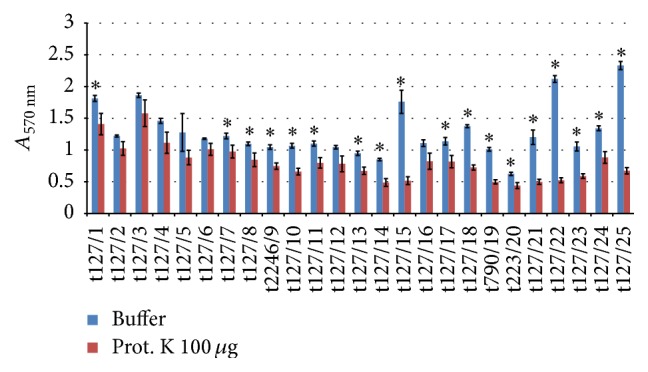
Dispersal of 48 h mature methicillin-resistant* Staphylococcus aureus* (MRSA) biofilms by proteinase K. The MRSA isolates are indicated on the *x*-axis; the biofilms matured for 16 h were treated by buffer alone (blue bar) or buffer containing 100 *μ*g/mL proteinase K (red bar). Bars represent the mean values ± standard error of the mean of at least three independent experiments. Asterisk (*∗*) indicates a *p* value of less than 0.05 between the treated group and corresponding control.

Biofilm formed from all of the isolates displayed a range of sensitivities to proteinase K (100 *μ*g/mL) (Figure 5). Isolate t127/6 showed only a 14% reduction in biofilm biomass, whereas isolate t127/22 showed strong dispersal of the biofilm (a 75% reduction). No significant biofilm dispersal was observed for isolates t127/2, t127/3, t127/4, t127/5, t127/6, t127/12, and t127/16; however, these isolates displayed a reduction in biomass of up to 30%. In contrast, isolates t127/11, t127/13, and t223/20 exhibited significant differences between their replicates, with a 29% reduction in biofilm formation by isolates t127/13 and t223/20, whereas isolate t127/11 showed only a 27% reduction relative to that of the control.

**Figure 6:**
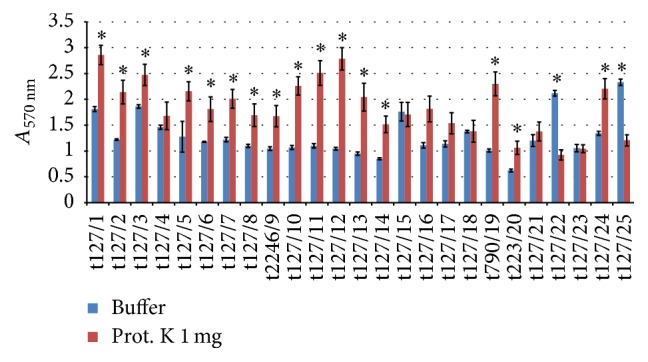
Dispersal of 48 h mature methicillin-resistant* Staphylococcus aureus* (MRSA) biofilms by proteinase K. The MRSA isolates are indicated on the *x*-axis; the biofilms matured for 16 h were treated by buffer alone (blue bar) or buffer containing 1 mg/mL proteinase K (red bar). Bars represent the mean values ± standard error of the mean of at least three independent experiments. Asterisk (*∗*) indicates a *p* value of less than 0.05 between the treated group and corresponding control.

Because proteinase K (100 *μ*g/mL) did not completely disperse the established biofilms, the experiments were repeated with a higher concentration of proteinase K (1 mg/mL). Interestingly, as shown in Figure 6, proteinase K at this concentration enhanced biofilm formation in the majority of the isolates tested, except for isolates t127/22 and t127/25, which showed reductions in biofilm biomass of 56% and 48%, respectively. Isolates t127/15, t127/18, and t127/23 seemed not to be affected by proteinase K at this concentration, in spite of showing sensitivity to proteinase K at the lower concentration of 100 *μ*g/mL.

**Figure 7:**
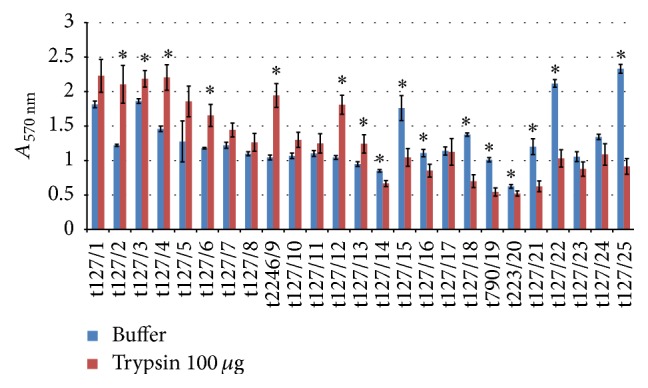
Dispersal of 48 h mature methicillin-resistant* Staphylococcus aureus* (MRSA) biofilms by trypsin. The MRSA isolates are indicated on the *x*-axis; biofilms matured for 16 h were treated by buffer alone (blue bar) or buffer containing 100 *μ*g/mL trypsin (red bar). Bars represent the mean values ± standard error of the mean of at least three independent experiments. Asterisk (*∗*) indicates a *p* value of less than 0.05 between the treated group and corresponding control.

**Figure 8:**
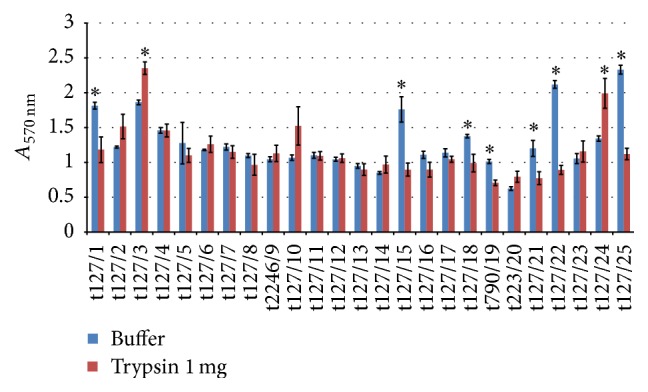
Dispersal of 48 h mature methicillin-resistant* Staphylococcus aureus* (MRSA) biofilms by trypsin. The MRSA isolates are indicated on the *x*-axis; biofilms matured for 16 h were treated by buffer alone (blue bar) or buffer containing 1 mg/mL trypsin (red bar). Bars represent the mean values ± standard error of the mean of at least three independent experiments. Asterisk (*∗*) indicates a *p* value of less than 0.05 between the treated group and corresponding control.

When trypsin (100 *μ*g/mL) was added to a 48 h established biofilm, some of the isolates displayed biofilm dispersion, whereas others displayed biofilm enhancement. As seen in Figure 7, isolates t127/15, t127/18, t223/19, t127/21, t127/22, and t127/25 showed a significant reduction in biofilm biomass (up to 60%) when compared to isolates t127/14, t127/16, t223/20, and t127/23, which displayed a reduction of no more than 23%. The remaining isolates showed biofilm enhancement in the presence of trypsin (100 *μ*g/mL). The experiments when repeated with a higher concentration of trypsin (1 mg/mL) (Figure 8) and isolates t127/1, t127/15, t127/18, t223/19, t123/21, t127/22, and t127/25 showed reductions in biofilm biomass of up to 57%. However, isolates t127/2 and t127/10 showed a noticeable but not significant increase in biofilm biomass compared with isolates t127/3 and t127/24. Interestingly, biofilm biomass increased with an increase in enzyme concentration for isolate t127/3, from 17% with 100 *μ*g/mL trypsin to 26% with 1 mg/mL trypsin, and for isolate t127/10, which increased from 21.6% to 42%.

**Figure 9:**
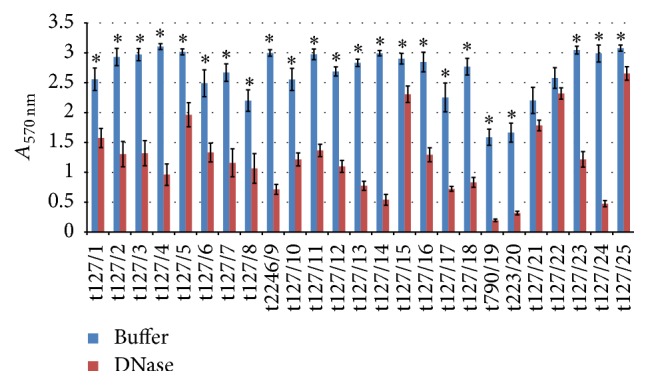
Dispersal of 48 h mature methicillin-resistant* Staphylococcus aureus* (MRSA) biofilms by DNase. The MRSA isolates are indicated on the *x*-axis; the biofilms matured for 16 h were treated by buffer alone (blue bar) or buffer containing 140 U/mL DNase (red bar). Bars represent the mean values ± standard error of the mean of at least three independent experiments. Asterisk (*∗*) indicates a *p* value of less than 0.05 between the treated group and corresponding control.

**Figure 10:**
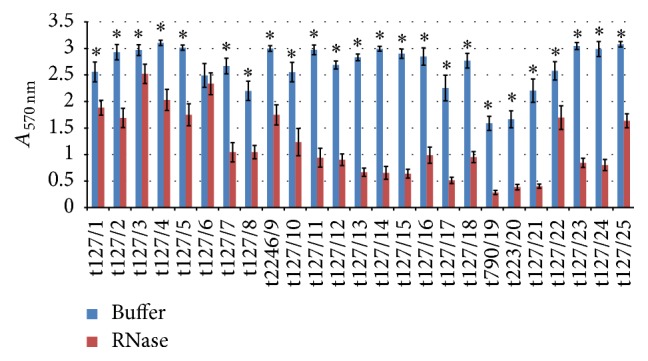
Dispersal of 48 h mature methicillin-resistant* Staphylococcus aureus* (MRSA) biofilms by RNase digestion. The MRSA isolates are indicated on the *x*-axis; the biofilms matured for 16 h were treated by buffer alone (blue bar) or buffer containing 100 *μ*g/mL RNase (red bar). Bars represent the mean values ± standard error of the mean of at least three independent experiments. Asterisk (*∗*) indicates a *p* value of less than 0.05 between the treated group and corresponding control.

Figure 9 shows that DNase reduced biofilm for the majority of isolates tested, with a loss in biofilm biomass of up to 84%, except for isolates t127/21 and t127/22, which showed less sensitivity to DNase, with 19% and 10% reductions in biofilm biomass with *p* values of 0.09 and 0.2, respectively. Similarly to this effect, biofilm biomass was moderately to highly sensitive to dispersal by RNase, as shown in Figure 10. The majority of isolates were highly sensitive, with biofilm reductions of up to 78% (*p* ≤ 0.009). On the other hand, isolates t127/1, t127/3, and t127/6 showed minimal reductions in biofilm biomass (26%, 15%, and 6%, resp.). This indicated that both eDNA and extracellular RNA (eRNA) were components of the biofilm matrix produced by all of these isolates.

**Figure 11:**
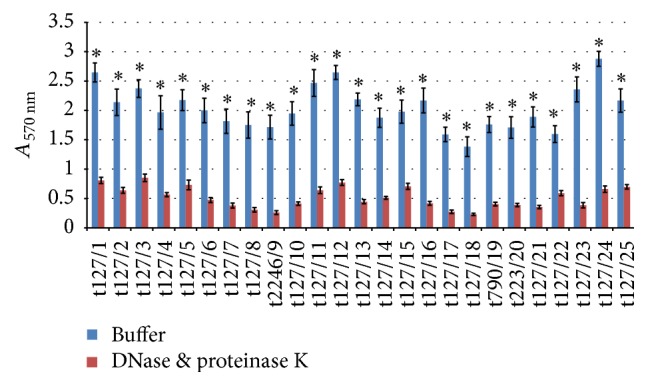
Dispersal of 48 h mature methicillin-resistant* Staphylococcus aureus* (MRSA) biofilms by DNase and proteinase K. The MRSA isolates are indicated on the *x*-axis; mature biofilms were treated by buffer alone (blue bar) or by consecutive DNase and proteinase K treatment (red bar). Bars represent the mean values ± standard error of the mean of at least three independent experiments. Asterisk (*∗*) indicates a *p* value of less than 0.05 between the treated group and corresponding control.

Many previous studies have shown that eDNA and proteins are main components of MRSA biofilms. Our study found that DNase I was a more effective biofilm inhibitor than proteinase K, but that neither dispersed biofilms completely. The maximum percentage biofilm dispersal by DNase was 84%, whereas, with proteinase K, this was 75%. To investigate whether DNase and proteinase K could complement each other to eliminate biofilms, 48 h established biofilms were treated consecutively with DNase and proteinase K treatment. As shown in Figure 11, the majority of isolates showed a significantly greater (*p* = 0.001) reduction in biofilms compared to that with DNase or proteinase K alone. However, isolates t127/14, t790/19, t223/20, and t127/24 showed more effective biofilm dispersal when treated with DNase alone, compared with either treatment with proteinase K alone or treatment with DNase followed by proteinase K.

### 4.5. Biofilm Adhesiveness and Cohesiveness

In previous experiments in this study, the emphasis was on detecting biofilm components. To investigate whether cell-to-surface adhesion and cell-to-cell cohesion can be affected by biofilms, the MRSA isolates were tested for these abilities. The isolates could be classified into two categories, depending on biofilm components found in this study. The first category comprised those isolates that formed PIA-independent biofilms, which included t127/2, t127/3, t127/5, t127/10, t127/11, t127/12, t127/13, t127/15, t127/16, t127/21, t127/23, and t127/24. The second category comprised isolates that formed PIA-dependent biofilms, which included t127/1, t127/4, t127/6, t127/7, t127/8, t2246/9, t127/14, t127/17, t127/18, t790/19, t223/20, t127/22, and t127/25, regardless of the exopolysaccharide quantity or whether the isolates possessed weak or moderate biofilm-forming abilities.

**Figure 12:**
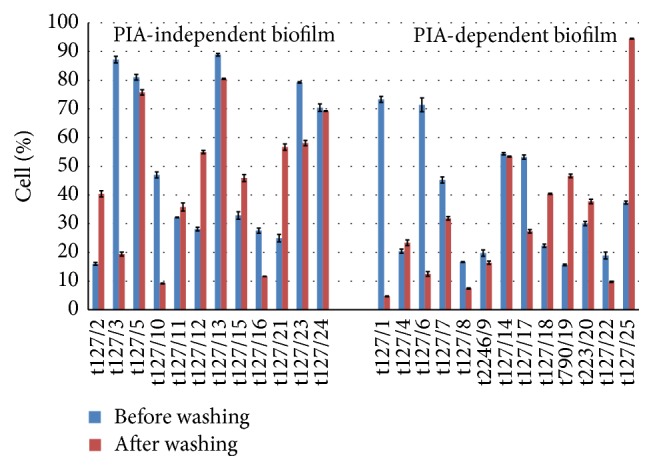
Biofilm adhesiveness assay for PIA-independent and PIA-dependent biofilms. The *x*-axis shows methicillin-resistant* Staphylococcus aureus* isolates before (blue bars) and after washing (red bars); the *y*-axis shows the percentage of cells that adhered to glass beads. Bars represent the mean values ± standard error of the mean of at least three independent experiments.

**Figure 13:**
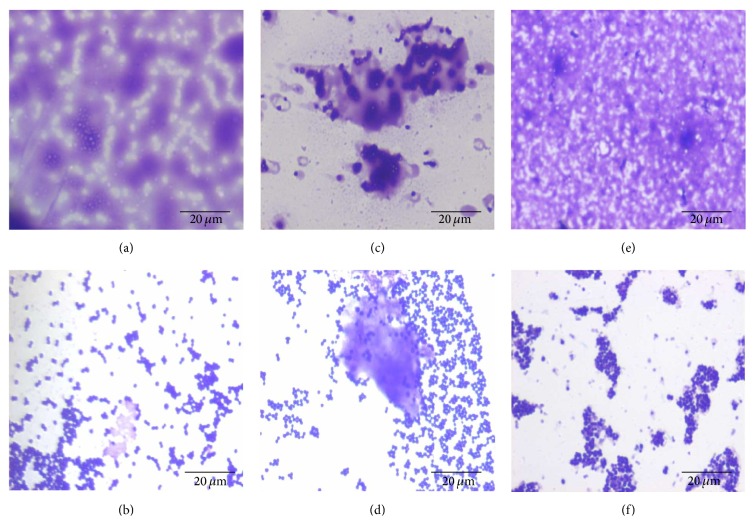
Images of methicillin-resistant* Staphylococcus aureus* biofilms stained with crystal violet and examined by light microscopy at a magnification 100x. (a) Unwashed and (b) washed cells of isolate t127/2; (c) unwashed and (d) washed cells of isolate t127/18; (e) unwashed and (f) washed cells of isolate t127/21. ((a) and (b)) Biofilms prevented crystal violet from penetrating cell walls. ((a), (b), and (c)) Cell clusters were completely enveloped by the biofilm matrix. (d) Large batches of biofilm that covered some cells. Scale bar = 20 *μ*m.

In the adhesion assay, the impact of biofilms on cell adhesion to the surface of glass beads was investigated using unwashed and washed bacteria. As shown in Figure 12, isolates t127/3, t127/5, t127/10, t127/13, t127/16, and t127/23 in the PIA-independent biofilm category exhibited increased adhesion to glass beads. Similarly, the PIA-dependent isolates t127/1, t127/6, t127/7, t127/8, t2249/9, t127/17, and t127/22 also showed increased adhesion to glass beads. There appeared to be no correlation between biofilm components and cell adhesiveness, as the washed cells of isolates t127/2, t127/11, t127/12, t127/15, and t127/21, which formed PIA-independent biofilms, and t127/4, t127/18, t790/19, t223/20, and t127/25, which formed PIA-dependent biofilms, appeared to have increased abilities to adhere to glass beads compared to those of unwashed cells.  Figure 13 shows that the EPS from cells that were only partially removed by the rinsing procedure did not always exhibit increased abilities of MRSA* spa* type t127 cells to adhere to surfaces.

Bacterial cohesiveness is shown in Table 2. The isolate t127, which formed a PIA-independent biofilm, showed aggregation of 13% to 47% compared to those isolates that formed PIA-dependent biofilms, which showed 6% to 54% aggregates. The isolates t2246, t790, and t223 displayed cell aggregation percentages of 38%, 23%, and 17%, respectively.

These results indicated no correlation between biofilm components and cell-to-cell associations within biofilms. Interestingly, isolates t127/3, t127/5, t127/10, t127/13, t127/16, and t127/23, which formed PIA-independent biofilms, and isolates t127/1, t127/6, t127/7, t127/8, t2246/9, t127/17, and t127/22, which formed PIA-dependent biofilms, showed a high percentage of adhesiveness in unwashed cells compared to percentage of cell aggregation as their ability to adhere onto glass beads after the washing process is reduced. In contrast, isolates t127/2, t127/11, t127/12, t127/15, and t127/21, which formed PIA-independent biofilms, and isolates t127/4, t127/18, t790/19, t223/20, and t127/25, with PIA-dependent biofilms, showed increased adhesion to glass beads after the washing process. Both the washed and unwashed PIA-independent biofilm of isolate t127/24 and PIA-dependent biofilm of isolate t127/14 showed similar cell adhesiveness and cohesiveness. The relationship between cell-to-surface adhesion and cell-to-cell cohesion within biofilms of MRSA isolates shall be addressed in a more intensive study.

## 5. Discussion

MRSA biofilms play a significant role in numerous chronic infections [35, 36]. To improve MRSA diagnostics, it is necessary to understand the biofilms that lead to chronic infections [37]. Although there have been many studies on the components of MRSA biofilms, very few of these studies have addressed the impact of biofilms on the adhesiveness and cohesiveness of bacterial cells [13, 14, 38–40].

The gene* spa* type t127 is frequently present community-acquired MRSA in the UK [41], as well as in the US [42]. Similarly, in this study, we found that the majority of MRSA isolates tested had* spa* type t127, with a small number having* spa* types t2246, t790, and t223. Based on a semiquantitative microwell plate assay, the majority of these isolates showed a moderate ability to produce biofilms. The production of slime on TSAG (Figure 3(a)), however, did not seem to be related to the adhesion strength of these biofilms on microwell plates.

Assessing biofilm dispersal is considered the main method to determine the components involved in biofilm formation. In our study, antibiofilm agents such as NaIO_4_ and extracellular enzymes were used to try to disperse mature biofilms of isolates t127, t2246, t790, and t223. These antibiofilm agents have been shown to eliminate biofilms from nonliving and living surfaces [43, 44]. However, it is important to consider the structures of the biofilms that are being targeted [45], as many of these agents differ in their effects on the various forms of biofilms produced by different bacterial species [14, 46, 47].

PIA/PNAG polymeric chains appear to be major constituents of many biofilms in both Gram-positive and Gram-negative pathogens [48]. NaIO_4_ can modify these polymeric chains by splitting the C3-C4 bonds on exopolysaccharide residues and oxidizing the carbons to yield vicinal hydroxyl groups [45]. Our study showed that NaIO_4_ had varying effects, from high to low levels of biofilm reduction for MRSA isolates related to clone t127. This could be of a result of the effects of NaIO_4_ on exopolysaccharides that are chemically identical in structure, but that have some differences in both the amount of acetates O-linked with succinate and acetylation levels of amino groups [32, 49]. In biofilms, the polysaccharides do not exist alone but appear either in association or segregated, interacting with a broad range of other molecular species, including DNA, proteins, and lipids [50]. As a consequence, depolymerisation of exopolysaccharides in response to NaIO_4_ varies depending on biofilm components. In our study, the colony morphologies of MRSA isolates, observed on Congo red agar, revealed different patterns of interaction between the exopolysaccharides (black colour) and proteins (red colour); some isolates produced smooth, black and red colonies and others produced mucoid red-black colonies with a red pellet that appeared to have melted inside (Figure 3(b)). Sager et al. [51] showed that NaIO_4_ had a stimulating influence on established biofilms of* Pasteurella pneumotropica.*

The exopolysaccharides present in bacterial capsules seemed to have a negative effect on biofilm production. For example, mutations in the capsule genes of* S. haemolyticus*,* Vibrio vulnificus*, and* Porphyromonas gingivalis* resulted in an increase in biofilm formation compared to the wild-type strains because of decreased capsular exopolysaccharide production [52–54]. NaIO_4_ seemed to enhance the production of biofilms, as indicated in Figure 4, by increasing the ability of some MRSA isolates related to clone t127 to produce biofilms. This could be the result of exopolysaccharides present in the capsules of bacteria being eliminated.

Protease treatment is known to disperse mature MRSA biofilms. Kumar Shukla and Rao [55] showed that proteinase K treatment impaired biofilm formation because of the absence of biofilm-associated protein (encoded by* Bap*) on the surface of* S. aureus* strain V329, but that it did not have any effect on strain M556, which lacked* Bap*. In this study, proteinase K and trypsin were used to determine whether proteins were components of mature biofilms. Proteinase K (100 *μ*g/mL) caused preformed biofilms to detach, but with dispersal percentages that were comparatively low for all 25 MRSA isolates tested. However, the majority of our isolates appeared to be sensitive to proteinase K (100 *μ*g/mL), consistent with the findings of previous studies that showed the high sensitivity of* S. aureus* biofilms to proteinase K [13, 14, 40, 45, 47]. Our results showed that in 48 h established biofilms, treatment with a high concentration of proteinase K (1 mg/mL) promoted biofilm formation by all of the isolates except t127/22 and t127/25.

Additionally, trypsin (100 *μ*g/mL) showed a variety of effects. In half of the isolates studied, including isolates related to clones t127 and t2246, trypsin treatment increased biofilm formation, whereas in the other half, including isolates related to clones t127, t790, and t223, it decreased biofilm biomass to varying degrees. Interestingly, trypsin (1 mg/mL) was able to partially remove biofilms of some isolates. However, the reason behind these inconsistent observations in the interactions between the two common proteases, trypsin and proteinase K, is not clear. The biofilms of some of isolates were efficiently removed by both proteases. According to Boles and Horswill [44], proteinase K inhibited biofilm formation and promoted the dispersal of established biofilms. Our results agreed with findings by Gilan and Sivan [56], who showed that proteinase K (1 mg/mL) treatment doubled the size of a* Rhodococcus ruber* C208 biofilm. Moreover, the biofilm seemed to be multilayered, mucoid, and more robust than that before treatment. However, the established biofilm was decreased by trypsin, with a monolayered, sparser structure resulting. We propose that a high concentration of proteinase K enhances autolysis of bacterial cells, thereby releasing extracellular DNA [57, 58].

eDNA is an important part of biofilm structure [59]. This was first discovered in* Pseudomonas aeruginosa* and then in other bacterial species [17, 60–63]. eDNA is released mainly through cell lysis [64–68] or is secreted from cells [63, 69, 70]. Biofilm formation has been reported to be blocked, or its morphology altered, by DNase I treatment of Gram-negative cells such as* Pseudomonas aeruginosa* and* Escherichia coli*, as well as Gram-positive cells such as* S. aureus*,* S. pneumonia*, and* L. monocytogenes* [59, 71, 72]. Our data shows that DNase I significantly affected the dispersal of biofilms in the majority of isolates tested. Consistent with this, Rice et al. [17] found that the structural stability of* S. aureus *biofilms depended on eDNA. Moreover, DNase I-induced degradation of eDNA resulted in a reduction in the biofilm.

Mulcahy et al. [73] suggested that eDNA not only increased biofilm stability but also its resistance to antibiotics. Our study showed that eRNA is also an important part of biofilms, as similar effects on established biofilms were observed in response to DNase I and RNase treatment (Figure 10). Nishimura et al. [74] showed the presence of eRNA in biofilms of the marine bacterium* Rhodovulum sulfidophilum*. Similarly, Gilan and Sivan [75] showed that applying RNase to cultures of* Rhodococcus ruber* strain C208 reduced biofilm formation. They also showed that the formation of biofilms was not increased by the addition of short fragments of DNA (*ca*. 300 and 500 bp) in C208 culture. Izano et al. [62] suggested that the size of the eDNA in* S. aureus* is important to the formation of biofilms, as different forms of nucleic acids play different roles in this process. eDNA seems to be important structural component of biofilms, whereas eRNA may be involved in regulating biofilm formation because of the significant size difference between these molecules.

To confirm the role of protein in biofilm formation, 48 h biofilms were first treated with DNase I and then by proteinase K in microwell plates. The results, shown in Figure 11, confirmed the significant roles played by both DNA and proteins in biofilm matrix formation. Our findings are consistent with an earlier report showing that MRSA biofilms decreased significantly in the presence of the two enzymes as compared to treatment with the individual enzymes alone [31]. This is further supported by the observation that autolysin (encoded by* Atl*) and fibronectin-binding proteins (encoded by* FnBP*) expression is a basic feature of the MRSA biofilm phenotype [13, 19].

Many studies have shown that biofilms are sessile communities of bacteria that precipitate and adhere to all surfaces [76, 77]. The architecture of a biofilm is dependent on cell-to-surface and cell-to-cell interactions [24, 78–80]. Figure 12 shows that biofilms of some MRSA isolates only weakly adhered to glass beads, whereas these same isolates strongly adhered to glass beads after extensive washing.

We speculate that slime layers on biofilms reduced the ability of the biofilms to adhere to glass beads. As shown in Figure 13, the washing process reduced the amount of slime present on the biofilms and increased the percentage of cells that aggregated. It is probable that after the washing process, some clusters of bacteria were still covered or surrounded by remnants of the polymer matrix, thereby increasing the adhesiveness of cells to glass beads. These findings are consistent with those of Gómez-Suárez et al. [81], who reported that the ability to adhere to solid surfaces was greater for nonbiofilmed* Pseudomonas aeruginosa* SG81R1 than for biofilmed* P. aeruginosa* SG81.

Our data showed a specific relationship between adhesiveness and cohesiveness of the MRSA biofilm isolates tested. When the percentage of cell-to-cell aggregates (Table 2) was higher than that of cell-to-surface aggregates in biofilms, the cells seemed to have an increased ability to attach to glass beads after washing. However, when the percentage of cell-to-cell aggregates was lower than that of cell-to-surface aggregates, the ability of the cells to attach to glass beads was reduced after washing (Figure 12). MRSA isolates in this study did not depend on static electricity and polymeric interactions to adhere to glass surfaces as proposed by Tsuneda et al. [24], as there was no correlation between the amount of EPS in the biofilms and cell adhesiveness. This could be because the majority of our isolates produced a moderate amount of biofilm. Moreover, there was no correlation between cell adhesiveness and PIA independence or dependence of the biofilms.

## 6. Conclusion

Based on the comparative analysis of biofilm extracellular matrices, it can be concluded that the tested biofilms consisted of nucleic acid-protein complexes, with or without exopolysaccharides. Different biofilm phenotypes were observed for the same MRSA clone. In addition, there seemed to be an association between cellular adhesiveness and cohesiveness of MRSA biofilms.

## Figures and Tables

**Table 1:** Quantification of biofilms formed by methicillin-resistant *Staphylococcus aureus* isolates by microwell plate assay. Biofilms were stained with 0.1% crystal violet solution after 48 h of incubation at 37°C. The values represent mean ± standard error of mean (SEM) for three independent replicates.

Isolates	Biofilm formation Mean ± SEM	Type of Biofilm
t127/1	3.246 ± 0.099	Moderate (++)
t127/2	3.248 ± 0.134	Moderate (++)
t127/3	3.071 ± 0.352	Moderate (++)
t127/4	3.245 ± 0.055	Moderate (++)
t127/5	3.226 ± 0.115	Moderate (++)
t127/6	3.256 ± 0.070	Moderate (++)
t127/7	3.121 ± 0.067	Moderate (++)
t127/8	2.942 ± 0.282	Moderate (++)
t2246/9	2.771 ± 0.425	Moderate (++)
t127/10	2.761 ± 0.438	Moderate (++)
t127/11	2.590 ± 0.448	Weak (+)
t127/12	3.114 ± 0.330	Moderate (++)
t127/13	2.409 ± 0.440	Weak (+)
t127/14	2.575 ± 0.729	Weak (+)
t127/15	3.166 ± 0.110	Moderate (++)
t127/16	2.696 ± 0.740	Moderate (++)
t127/17	2.616 ± 0.951	Weak (+)
t127/18	2.083 ± 0.617	Moderate (++)
t790/19	2.879 ± 0.618	Moderate (++)
t223/20	2.735 ± 0.750	Weak (+)
t127/21	1.916 ± 0.970	Weak (+)
t127/22	1.219 ± 0.406	Moderate (++)
t127/23	2.884 ± 0.548	Moderate (++)
t127/24	2.696 ± 0.533	Moderate (++)
t127/25	3.257 ± 0.095	Moderate (++)

**Table 2:** Percentage of methicillin-resistant *Staphylococcus aureus* isolates that aggregated after mechanical disruption of the biofilms.

Isolates	Aggregation%
t127/1	36
t127/2	17
t127/3	28
t127/4	47
t127/5	41
t127/6	32
t127/7	6
t127/8	12
t2246/9	38
t127/10	13
t127/11	24
t127/12	20
t127/13	27
t127/14	34
t127/15	34
t127/16	20
t127/17	19
t127/18	34
t790/19	23
t223/20	17
t127/21	47
t127/22	18
t127/23	47
t127/24	34
t127/25	58
